# Protective effect of N-acetylcysteine against cisplatin ototoxicity in rats: a study with hearing tests and scanning electron microscopy^☆^

**DOI:** 10.1016/j.bjorl.2018.08.002

**Published:** 2018-09-14

**Authors:** Mehmet Akif Somdaş, İnayet Güntürk, Esra Balcıoğlu, Deniz Avcı, Cevat Yazıcı, Saim Özdamar

**Affiliations:** a Erciyes University, Faculty of Medicine, Department of Otorhinolaryngology, Kayseri, Turkey; b Erciyes University, School of Medicine, Department of Medical Biochemistry, Kayseri, Turkey; c Erciyes University, School of Medicine, Department of Histology and Embryology, Kayseri, Turkey; d Patnos State Hospital, Department of Otorhinolaryngology, Ağrı, Turkey

**Keywords:** Cisplatin, Electron microscopy, Hearing tests, N-acetylcysteine, Ototoxicity, Cisplatina, Microscópio eletrônico, Testes de audição, N-acetylcysteine, Ototoxicidade

## Abstract

**Introduction:**

Ototoxicity is a health problem appearing after powerful treatments in serious health conditions. It is sometimes inevitable when treatment of the serious disease is required. Cisplatin is an antineoplastic agent which was investigated previously to reveal increased nitrogen and reactive oxygen radicals that damages hair cells, resulting in ototoxicity. N-acetylcysteine, previously shown to decrease ototoxicity caused by different agents, is known to be a powerful *in vitro* antioxidant. Probably N-acetylcysteine, in addition to its antioxidant effect, blocks a cascade where reactive oxygen species result in apoptosis in the cochlea.

**Objectives:**

The possible preventive effect of N-acetylcysteine in cisplatin ototoxicity was studied with auditory brain stem responses, otoacoustic emissions, and histopathological investigation of the cochlea in a scanning electron microscopy.

**Methods:**

This study was conducted on 21 Wistar Albino rats in four groups. 1 mL/kg/day three times in total intraperitoneal (i.p.) Saline (*n* = 5), 500 mg/kg/day i.p. three times in total N-acetylcysteine (*n* = 5), i.p. 15 mg/kg cisplatin alone (single dose) (*n* = 5) and i.p. 15 mg/kg cisplatin plus 500 mg/kg/day N-acetylcysteine (*n* = 6) were administered. The rats were anesthetized to study the hearing tests before and after the experiment. The rats were sacrificed to investigate the cochleas by scanning electron microscopy.

**Results:**

Auditory brain stem responses and otoacoustic emissions values were attenuated in the cisplatin group. The group that received N-acetylcysteine in addition to cisplatin had better auditory brain stem responses thresholds and otoacoustic emissions. The samples obtained from the cisplatin group showed surface irregularities, degeneration areas, and total or partial severe stereocilia losses. The changes were milder in the cisplatin + N-acetylcysteine group.

**Conclusion:**

Cisplatin ototoxicity can be detected by auditory brain stem responses and otoacoustic emissions testing in rats. N-acetylcysteine may protect the cochlear cells from histopathological changes. We concluded that N-acetylcysteine given 4 h after cisplatin injection has a potential otoprotective effect against cisplatin ototoxicity. which suggests it could be used in clinical trials.

## Resumo

**Introdução:**

A ototoxicidade é um problema que pode ocorrer após certos tipos de tratamentos para condições graves de saúde. Às vezes é inevitável quando o tratamento da doença é necessário. A cisplatina é um agente antineoplásico cujo uso em pesquisas anteriores demonstrou aumentar os radicais livres de nitrogênio e espécies reativas de oxigênio que danificam as células ciliadas e resultam em ototoxicidade. Por outro lado, a N-acetilcisteína, que já demonstrou diminuir a ototoxicidade causada por diferentes agentes, é conhecida por ser um potente antioxidante in vitro. Provavelmente a N-acetilcisteína, além de seu efeito antioxidante, bloqueia uma cascata onde espécies reativas de oxigênio resultam em apoptose na cóclea.

**Objetivos:**

Estudar o possível efeito preventivo da N-acetilcisteína na ototoxicidade por cisplatina por meio de potencial evocado auditivo de tronco encefálico, emissões otoacústicas e investigação histopatológica da cóclea por microscopia eletrônica de varredura.

**Método:**

Este estudo foi realizado em 21 ratos albinos Wistar, separados em quatro grupos. Foram administrados: 1 mL/kg/dia intraperitoneal (i.p.) de solução salina (n = 5), três vezes no total; 500 mg/kg/dia i.p. de N-acetilcisteína (n = 5), três vezes no total; 15 mg/kg i.p. (dose única) somente de cisplatina (n = 5) e 15 mg/kg i.p. de cisplatina e 500 mg/kg/dia i.p. de N-acetilcisteína (n = 6). Os ratos foram anestesiados para estudo dos testes auditivos antes e depois do experimento. Os ratos foram sacrificados para investigação da cóclea por microscopia eletrônica de varredura.

**Resultados:**

Os potenciais evocados auditivos de tronco encefálico e os valores das emissões otoacústicas estavam atenuados no grupo cisplatina. O grupo que recebeu N-acetilcisteína além da cisplatina apresentou melhores limiares de respostas auditivas do tronco encefálico e emissões otoacústicas. As amostras obtidas do grupo cisplatina apresentaram irregularidades de superfície, áreas de degeneração, com perdas graves totais ou parciais de estereocílios. As alterações foram mais leves no grupo cisplatina + N-acetilcisteína.

**Conclusão:**

A ototoxicidade por cisplatina pode ser detectada por meio de potenciais evocados auditivos de tronco encefálico e pelo teste de emissões otoacústicas em ratos. A N-acetilcisteína pode proteger as células cocleares contra alterações histopatológicas. Concluímos que a N-acetilcisteína administrada 4 horas após a injeção de cisplatina tem potencial efeito otoprotetor contra a ototoxicidade por cisplatina e pode ser utilizada em ensaios clínicos.

## Introduction

Ototoxicity is a health problem appearing after powerful treatments in serious health conditions.^1^, ^2^, ^3^ It is sometimes inevitable when treatment of the serious disease is required; such as in cancer survivors. For cancer patients, Cisplatin is a common antineoplastic agent that was investigated previously to reveal increased nitrogen and reactive oxygen radicals that damages hair cells resulting in ototoxicity.^3^, ^4^, ^5^, ^6^, ^7^, ^8^

There are a few agents, including sodium thiosulfate, amifostine, D-methionin, vitamin E, dexamethasone, salicilates, neurotropins, flunarisine, lipoic acid, ebselen, diethyldithiocarbamate and 4-methylthiobensoic acid that claim to prevent ototoxicity.^9^, ^10^, ^11^, ^12^, ^13^, ^14^, ^15^ N-acetylcysteine (NAC), previously shown to decrease ototoxicity caused by different agents, is known to be a powerful *in vitro* antioxidant.^16^, ^17^, ^18^

Ototoxicity is mediated through reactive oxygen species, which results in cell death.^1^, ^2^, ^3^ In contrast, N-acetylcysteine is an antioxidant, which originally is a mucolytic for pulmonary treatment. However, it is also used for the diseases of the lungs, liver, heart and kidney in order to treat their toxic and ischemic injuries.^19^ For example, NAC improves renal hemodynamics in rats with cisplatin-induced nephrotoxicity.^20^ Probably NAC, in addition to its antioxidant effect, blocks a cascade where reactive oxygen species result in apoptosis in the cochlea.^21^

Permanent ototoxicity is a disabling condition that could further isolate the patient from the environment, in addition to the devastating effects of primary disease. Detection by Otoacoustic Emissions (OAEs) and Auditory Brainstem Responses (ABRs) and prevention of permanency by NAC would be very beneficial for the patient who is already dealing with the primary disease.

The purpose of this study is to evaluate the possible protective properties of a potent antioxidant NAC against cisplatin ototoxicity which occurs through free radicals. NAC may contribute to the literature to prevent ototoxicity in cisplatin chemotherapy because NAC can be used in humans.

## Methods

The study was accepted by Erciyes University Local Ethics Committee for Animal Experiments (HADYEK) (n° 16/147). Experiments were performed in Animal Laboratory. This is a prospective, controlled animal study about cisplatin ototoxicity.

A total of 21 male Wistar Albino 5 month-old rats with an average weight of 300–350 g, which were given a standard laboratory diet in the experiments, were studied. All rats were kept in cages in the same room and under the same environmental conditions, namely in a room which was illuminated and darkened for 12/12 h cycles at a temperature of 22 °C ± 3 with a background noise level of under 50 dB. The rats were fed ad libitum.

Initially, each rat was anesthetized with intraperitoneal (i.p.) ketamine (40 mg/kg) and xylazine (5 mg/kg). Following the anesthesia, the ear canals and tympanic membranes of each rat were examined with otomicroscopic inspection and no pathological finding were noted. Distortion Product Otoacoustic Emissions (DPOAEs) and ABRs were performed for both ears of each animal for baseline hearing threshold evaluation. During the experiments, three rats belonging to the control group, cisplatin group and NAC group and two rats belonging to the cisplatin + NAC group did not recover from anesthesia and were excluded from the study; therefore, new rats were included instead of dead animals in the study. In conclusion, 42 functionally normal ears of 21 rats were included in the study.

### Preliminary trial

First, a preliminary trial for ototoxicity on 20 rats was conducted. Cisplatin was administered to 15 Wistar Albino rats and ototoxicity was determined with decreament in. Saline was administered to 5 rats. Wistar Albino rats were administered cisplatin, first 5 mg/kg, followed by 10 mg/kg and 15 mg/kg until resultant ototoxicity. 15 mg/kg cisplatin dose was considered ototoxic with OAE and ABR tests. Thus 15 mg/kg cisplatin was used in the study.

### Formation of groups and experimental procedures

The subjects were randomized into 4 distinct groups as follows (Table 1).

**Table 1:** Distribution characteristics of all groups.

Group	Treatment protocol
Group 1 (*n* = 5)	1 mL/kg saline intraperitoneal (i.p.)
Group 2 (*n* = 5)	NAC 500 mg/kg (i.p.)
Group 3 (*n* = 5)	Cisplatin 15 mg/kg (i.p.)
Group 4 (*n* = 6)	Cisplatin 15 mg/kg + NAC 500 mg/kg (i.p.)

The rats in the third and fourth groups were administered cisplatin only once during the study. In the forth group NAC was administered three times in total (total dose 1500 mg/kg) on the first day 4 h after cisplatin, and on the second day and third days. At days 0 and 7, the subjects underwent anesthesia followed by OAEs and ABRs evaluation for hearing functions and the results were recorded.

After all injections and measurements were accomplished, the rats were sacrificed using high-dose anesthetic administration and the cochleae were harvested and fixed in formol solution for histopathological studies.

### Otoacoustic emission measurement

Signal to Noise Ratio (SNR) values, which were calculated by subtracting the background noise level from the DPOAE measurements in Decibel (dB), were used for interpretation of the test results. OAE system and neonatal probes were used for DPOAE screening. The f2/f1 ratio was fixated to 1.22, and L1–L2 difference was adjusted to 10 dB Sound Pressure Level (SPL) (L1 = 70 dB SPL; L2 = 60 dB SPL). DPOAEs were evaluated at the tones equal to 2f1-f2, and generated at the frequencies corresponding to the geometric mean of f1 and f2. SNR values were recorded from both ears on days 0 and 7, and at 2000, 3000, 4000, 6000, and 8000 Hz.

### ABR measurement

ABR responses were recorded by electrode needles subdermally located, with the active electrode at vertex, ground electrode on the glabella and reference electrodes on the right and left mastoid fields. Clicks were harnessed as auditory stimuli with pursuing regulations: bandpass filters of 100–3000 Hz and a repetition ratio of 21 s. The ABR sill was described on the fifth undulation. The sill was identified by launching at 70 dB, and when an appropriate wave form was obtained, by decreasing the volume by 20 dB each time. If an appropriate wave form did not occur, 90 dB was tried. When the wave was about to disappear, 10 dB decrements were instituted. The volume of the last wave, before it disappeared, is the threshold if repeatability was borne out and the sill identification was improved over two trials.

### Histopathological evaluation

The middle segment of cochleae was removed from the rats under xylazine and ketamine anesthesia within 7 days after the application. After removal of the bone tissue enveloping the internal auditory canal, the cochlear lumen was fixed in 2.5% glutaraldehyde for 48 h for the electron microscopic examination. Postfixation was performed with 1% osmium tetroxide (OsO_4_) and treated with increasing series of acetone (50%, 70%, 80%, 90%, 100%, 100%, 100%) for 15 min and dried with critical point dryer in a liquid CO_2_. The samples were placed on metal blocks and coated with gold-palladium at a thickness of 18–20 nm using a Sputter coater device and examined with LEO 440 scanning electron microscope in secondary electron mode at 15 kV.

### Statistical analysis

SPSS for Windows 16.0 was harnessed for investigating the findings of this experiment. For statistical analysis, the variables were expressed and used as number (*n*), percentage (%), and mean ± standard deviation. Shapiro–Wilk test, Q-Q, and histograms were used for assessment of the normality of the data. Comparisons were made by using two-way repeated measures analysis of variance. One-way Kruskal–Wallis test was used for comparing DPOAE outcomes between groups before and after drug application. Bonferroni test was implemented for multiple comparisons. Intra-group comparisons were utilized from Wilcoxon analysis. Values of *p* < 0.05 were accepted as statistically meaningful.

## Results

### Evaluation of DPOAE and ABR results

Among all groups and intra-group, baseline DPOAE values were not markedly different. After cisplatin application, DPOAE values and ABR tresholds were attenuated. There are statistically meaningful changes on ABR tresholds before and after cisplatin administration (*p* < 0.05) (Table 2). Regarding saline group there was no significant change in hearing tresholds. NAC alone exhibited significant change in hearing thresholds, in the opposite direction after treatment. ABR tresholds of Cisplatin plus NAC group were significantly attenuated, but this was less than Cisplatin alone group (Table 2). There are statistically meaningful changes on ABR comparisons between group 1–3, 2–3, 2–4 and 3–4 (*p* < 0.05) (Table 3). The cisplatin treatment markedly decreased DPOAE responses in all frequencies (Table 4). Changes in DPOAE responds in the other three groups were not significant. The significant changes on ABR thresholds between pretreatment and posttreatment in NAC group is not ototoxicity. There was improvement in hearings of the NAC group.

**Table 2:** Pretreatment and post-treatment auditory brainstem response (ABR) thresholds in the groups (mean value).

Group	Pre-treatment	Post-treatment	*p*
Control group	26 ± 14.29	23 ± 10.59	0.083
NAC group	28 ± 7.88	17 ± 11.5	0.004^a^
Cisplatin group	26 ± 10.74	37 ± 11.59	0.005^a^
Cisplatin + NAC group	−11.25 ± 17.72	−5 ± 15.81	0.006^a^

Intra group comparisons were utilized from Wilcoxon Signed Ranks Test.

a Post-treatment statistically significant.

**Table 3:** Post-treatment auditory brainstem response (ABR) comparisons between the groups.

Groups	Test statistics	Standard error	*p*
1–2	−7.5	5.345	0.963
1–3	19.150	5.345	0.002^a^
1–4	12.867	5.117	0.072
2–3	26.650	5.345	0.000^a^
2–4	20.367	5.117	0.000^a^
3–4	25.350	5.180	0.000^a^

1, Control group; 2, NAC group; 3, Cisplatin group; 4, Cisplatin + NAC group.

Kruskal–Wallis test were used for comparing ABR outcomes between groups.

a Statistically meaning.

**Table 4:** Pretreatment and post-treatment distortion product otoacoustic emission (DPOAE) responses in all frequencies (mean SNR, S, minimum and maximum values, *p*).

	Saline				NAC				Cisplatin				Cisplatin + NAC			
	SNR	A/B	S	*p*	SNR	A/B	S	*p*	SNR	A/B	S	*p*	SNR	A/B	S	*p*
Pre 2000	1.56	−8/7	5.0		−0.76	−9/6	5.4		1.31	−9/6	4.2		6.85	−4/12	3.8	
Post 2000	2.13	−8/14	6.8	0.959	−2.77	−14/6	5.8	0.44	−4.9	−13/2	5.2	0.03^a^	6.33	−3/12	4.3	1.0
Pre 3000	7.92	3/10	1.9		5.1	−4/11	5.5		6.28	4/7	0.8		12.55	6/21	5.3	
Post 3000	5.32	1/10	3.1	0.057	5.69	0/13	3.8	0.95	4.58	0/6	2.3	0.00^a^	11.71	1/21	5.8	0.75
Pre 4000	6.63	0/18	5.2		6.65	−2/15	5.2		4	−5/16	5.5		18.28	6/29	9	
Post 4000	5.66	−1/14	4.9	0.333	3.87	−7/21	8.6	0.20	0.93	−4/7	4	0.04^a^	15.96	6/27	6.7	0.58
Pre 6000	10.53	1/1	8.7		6.14	−12/32	12		2.73	−5/16	7.2		21.91	3/34	11.6	
Post 6000	5.36	27/8	2.2	0.074	7.23	−5/22	9.6	0.95	−3.74	−15/6	7.1	0.01^a^	18.16	−2/33	11.3	0.15
Pre 8000	11.37	0/−3	10		14.06	−11/38	14		2.66	−4/10	5.2		25.69	−5/46	16.2	
Post 8000	8.91	32/23	7.7	0.059	4.56	−14/25	11	0.00^a^	−4.53	−17/6	8.3	0.01^a^	17.95	−11/43	19.3	0.02^a^

SNR, signal noise ratio; S, standard deviation; A, minimum value; B, maximum value.

Intra group comparisons were utilized from Wilcoxon Signed Ranks Test.

a Statistically meaning.

### Histopathological results

**Figure 1:**
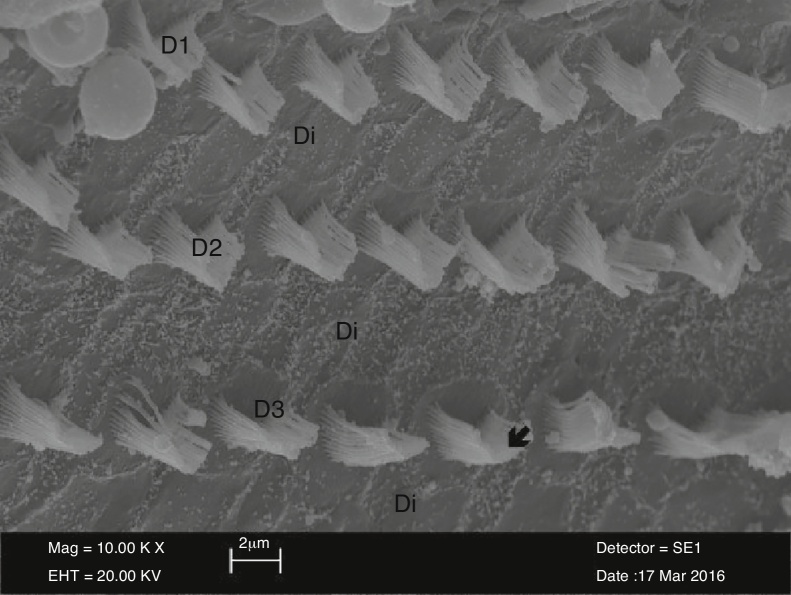
The rows of outer hair cells (D1-D2-D3) and the supporting Deiter cells (Di) and regularly organized stereocilia (↙) view in the control group (10,000×).

The outer hair cells in the control group were arranged in U or V-shape. There was a gradual increase in the length of stereocilia located in the outer surface, compared to those located in the inner surface. The stereocilia on the surface of the cells in the control group were arranged individually and regularly reflect the absorption and secretion functions of these cells (Fig. 1).

**Figure 2:**
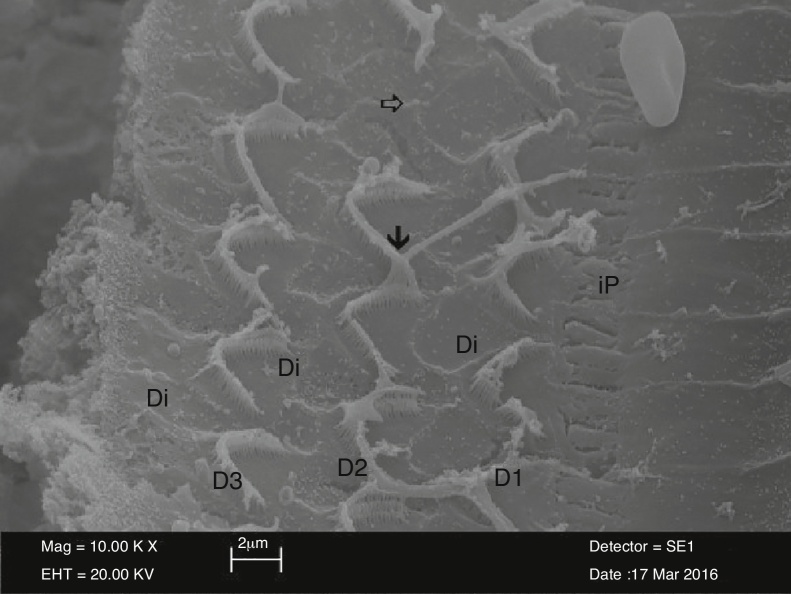
SEM view of outer hair cell structures (D1-D2-D3) surrounded by Deiter cells (Di) in the NAC group. The view of the areas of adhesions in apical section of stereocilia (↓) and the areas of loss in outer hair cell (⇒) İP, İnner Pillar cell (10,000×).

In electron microscopic examination of the surface morphology Organ of Corti in the NAC groups, stereocilia in the outer hair cells were similar to the control group and their U or V-shapes were preserved. However, the apical sections of stereocilia of these cells were strictly adhered to each other. Each stereocilium was unable to be differentiated due to these adhesions. In addition, structural irregularities and disruptions were observed in stereocilia. Similar to the control group, outer hair cells preserved their three-layer organization. However, there were adhesions and union between U or V-shape stereocilia in the NAC group, although there was no link between stereocilia located in the upper sections of the outer hair cells and stereocilia of the neighboring outer hair cells in the control group. There was a partial loss of stereocilia and total cell loss in successive outer hair cells (Fig. 2).

**Figure 3:**
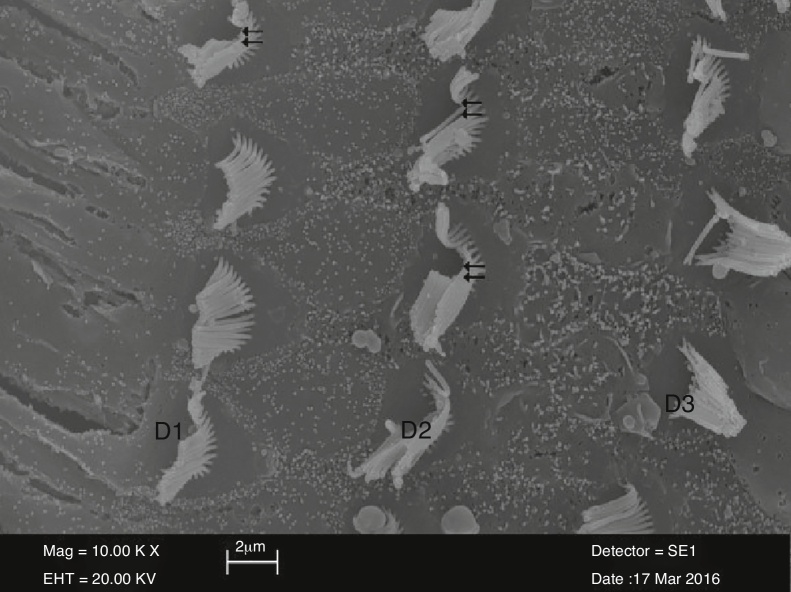
View of irregular stereocilia (D1-D2-D3) of the outer hair cells (⇇) in the cisplatin group (10,000×).

**Figure 4:**
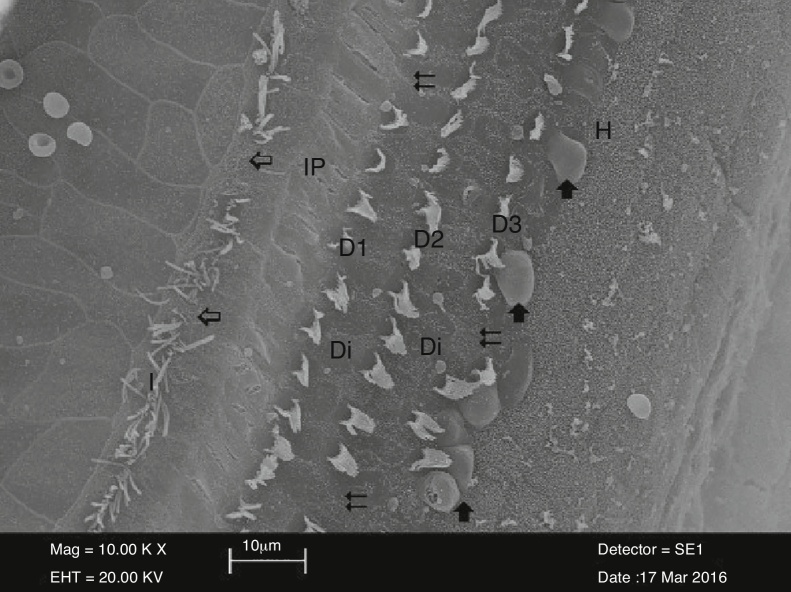
The area of partial loss (⇐) in stereocilia of inner hair cells (İ) and complete loss (⇇) in outer hair cells (D1-D2-D3) in the cisplatin group. View of balloon-like protrusions () in outer hair cells adjacent to Deiter cells (Di). İP, İnner Pillar cell; H, Hensen cell (3000×).

There were dissociations, irregularities, and curling between stereocilia in the examination of surface morphology Organ of Corti in the cisplatin group. Also, disruptions were observed in U or V-shape arrangement of the outer hair cells. In particular, there was a total loss or partial loss of stereocilia and areas of degeneration in three arrays of outer hair cells. There was also a wide range of areas of cellular deformation in the inner hair cells (Fig. 3). Balloon-like protrusions in the structure of the outer hair cells adjacent to Hensen's cells were also observed (Fig. 4).

**Figure 5:**
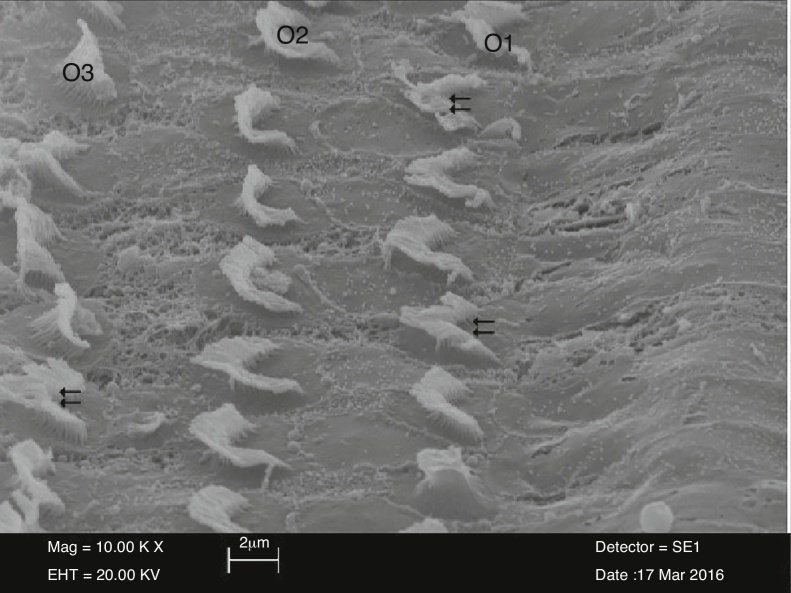
View of irregular and adhered stereocilia (⇇) of outer hair cells (O1-O2-O3) in the cisplatin plus NAC group (10,000×).

In cisplatin plus NAC group, outer hair cells were similar to those in the control group and preserved their U or V-shapes. However, there were adhesions, although not as severe as in the NAC group, in stereocilia located in the upper section of the outer hair cells. There was a total or partial loss of stereocilia between the outer hair cells organized in three layers. In addition, there were areas of degeneration in stereocilia (Fig. 5).

## Discussion

Different aspects of ototoxicity and preventive agents have been previously studied.^9^, ^10^, ^11^, ^12^, ^13^, ^14^, ^15^ Although there are other methods for detection of ototoxicity including postmortem histopathology, surveillance with OAEs and ABRs is a reliable way. Decrement in OAE produced by outer hair cells is the evidence of ototoxicity which was previously studied.^5^, ^7^, ^17^

NAC decreases hydrogen peroxide and increases cellular glutathione, thus known to reduce cisplatin ototoxicity.^22^, ^23^ NAC might exert osteogenic activity via increased glutathione synthesis.^24^ No osteogenesis was observed after the use of NAC in this study. But there is a debate that NAC can also decrease the antitumor effect since it is known to interact with cisplatin molecule. To prevent this attenuation in antitumor effect NAC was introduced via a totally different route, i.e. transtympani, which insured that two molecules do not interact.^25^ Transtympanic introduction of different protective molecules, e.g. sodium thiosulfate was also tried by others.^26^ To solve the interaction problem, Muldoon et al.^27^ introduced NAC 4 h after chemotherapy, and claimed that NAC chemoprotection did not alter cisplatin therapy, if delayed until 4 h after chemotherapy. They also claimed that this kind of protocol prevents ototoxicity. To test this in our current study, we also introduced the protective NAC 4 h after cisplatin injection, which turned out to reduce ototoxic effect cisplatin. While OAEs and ABRs were decreased in cisplatin group, NAC given 4 h after cisplatin did not alter OAE and ABR. We were able to clearly detect ototoxicity with these measurements in vivo. The cisplatin group revealed the worst results in OAE and ABR tests. OAE and ABR were not altered in the group that received NAC, meaning NAC did not affect hearing by itself. Low et al.^18^ evaluated NAC administered 72 h after radiation and claimed to see less oxygen radicals in the inner ear, which results in less apoptosis cochlea. This is similar to the mentality that we tested in the current study. In animal fertility studies, no adverse affects were reported at doses up to 250 mg/kg NAC and no teratogenic effects were observed at doses as high as 2000 mg/kg NAC.^28^ Duan et al.^29^ observed that a cumulative dose of NAC (1750 mg/kg), administered before and after impulse noise trauma, resulted in a greater permanent threshold shift and more inner hair cells loss compared to control animals. Instead, a cumulative dose of 1050 mg/kg, over 5 days, resulted in significant protection against impulse noise. In another experiment, two groups of animals were treated with the two different cumulative doses and were not exposed to acoustic trauma: these animals did not show any threshold shift. Thus, although NAC alone is not toxic for the cochlea, dosage of the drug is critical to elicit its protective effect. Fetoni et al.^30^ used a dose of 500 mg/kg i.p. administered immediately after noise exposure and then during the following two days (cumulative dose of 1500 mg/kg). So we used a dose of 500 mg/kg i.p. NAC. We did not observe a toxic effect of NAC.

As Okur et al.^22^ revealed that carboplatin ototoxicity increased nitric oxide levels and N-acetylcysteine prevented NO production, there may be different mechanisms about ototoxicity and prevention.

Regarding otopreservation toward antineoplastic drugs, particularly cisplatin, Church et al.^31^ found an electrophysiological practice of encephalic-induced potential in hamsters, preservation by sodium thiosulfate and diethyldimethylthiocarbamate, and they did not find influential protection against amifostine and fosfomycin. Kaltenbach et al.^32^ investigated the same drugs, now associated with structural evaluation by electron microscopy and brainstem induced potential. They determined 91% renovation of the outer hair cells with sodium thiosulfate, 68% with diethyldimethylcarbamate, 52% with fosfomycin and 45% with amifostine.

Fetoni et al.^30^ revealed that outer hair cells disappeared and disarrayed outer hair cell stereocilia bundles were observed. In contrast, noise exposure plus NAC showed only a moderate outer hair cell loss in the same regions and stereocilia were normal.

To our knowledge, combined audiological and histopathological findings regarding cisplatin ototoxicity and protection by NAC have not been previously published. We have shown that the ABR and OAE values and the numbers of damaged cells did not markedly change in the group receiving cisplatin and NAC, in which those receiving cisplatin alone markedly changed. Histopathological findings in the cochlea were also similar to the audiological findings. Electron microscopy showed outer hair cell loss in cisplatin ototoxicity. We concluded that cisplatin ototoxicity might be prevented using NAC in rats.

In conclusion, there was an increased length of hair cells from the basement to the apex in the cochlear structure. Based on these findings, the length of the inner and outer hair cells were unable to be compared between the experimental group and the control group. Therefore, we conclude that hearing impairment may develop as a result of problems in K^+^ flow caused by irregularities in tight junctions and desmosomes in stereocilia or blockade of channels by cisplatin, considering the fact that the sensory information is transported from the outer hair cells to the auditory canals.

Considering its effective dose, timing, and method of administration, NAC may serve as a valuable antioxidant agent for minimizing the ototoxicity of not only cisplatin, but also other substances. Other similar studies are necessary to support the delayed introduction of the protective agent.

## Conclusion

NAC does not exert any adverse effects on the inner ear when used alone. In our experimental model, cisplatin successfully triggered ototoxicity, which is evident from the decreases in DPOAE and ABR results and morphological findings; and NAC shows evident signals protection against cisplatin ototoxicity on the seventh day after application. NAC given 4 h after cisplatin injection prevented negative histopathological findings and the attenuation of OAE and ABR thresholds caused by cisplatin.

## Conflicts of interest

The authors declare no conflicts of interest.
